# Assessment of oral toxicity and safety profile of cyanidin: acute and subacute studies on anthocyanin

**DOI:** 10.2144/fsoa-2023-0322

**Published:** 2024-05-20

**Authors:** Swathi Suresh, Chitra Vellapandian

**Affiliations:** 1 Department of Pharmacology, SRM College of Pharmacy, SRM Institute of Science & Technology, Kattankulathur, Chengalpattu, Tamil Nadu, 603203, India; 2 Dean, SRM College of Pharmacy, SRM Institute of Science & Technology, Kattankulathur, Chengalpattu,Tamil Nadu, 603203, India

**Keywords:** acute, anthocyanin, cyanidin, OECD, oral toxicity, subacute

## Abstract

**Aim:** Purified anthocyanins lack a detailed safety profile, prompting the need for comprehensive oral toxicity research. **Materials & methods:** Sprague-Dawley rats aged 8 weeks received 300 mg/kg cyanidin orally for 14 days in acute toxicity (OECD 423). In the subacute study (OECD 407), adult SD rats were administered 7.5, 15 and 30 mg/kg/day cyanidin orally for 28 days. **Results:** Acute toxicity indicated an LD50 exceeding 300 mg/kg/day without adverse effects. Subacute toxicity at 7.5–30 mg/kg/day showed well-tolerated responses in both genders. No significant alterations in organ weights, hematological parameters, liver/kidney functions or adverse histopathological findings were observed. **Conclusion:** Oral cyanidin administration demonstrated high safety and tolerance in rats, establishing a NOAEL at 30 mg/kg/day, affirming cyanidin's safety for oral use.

## Plain language summary

Anthocyanins, natural pigments found in fruits and vegetables, lack a detailed safety profile. This study investigated the oral toxicity of cyanidin, a common anthocyanin. Acute toxicity testing in rats showed no adverse effects at doses up to 300 mg/kg. In the subacute study, doses of 7.5–30 mg/kg/day over 28 days were well tolerated, with no significant negative effects on organ function or histopathology. The findings suggest that cyanidin is safe for oral use in rats, with a No Observed Adverse Effect Level (NOAEL) established at 30 mg/kg/day.

## Tweetable abstract

Rat studies reveal cyanidin, a common anthocyanin, shows high oral safety at doses up to 300 mg/kg/day, paving the way for safer dietary supplement use. #Toxicology #SafetyResearch

## Abstract (plain language summary)

Anthocyanins, natural pigments in fruits and vegetables, lack detailed safety profiles.

Comprehensive oral toxicity research is needed to assess the safety of purified anthocyanins.

Acute toxicity testing involved administering 300 mg/kg cyanidin orally to Sprague-Dawley rats for 14 days.

subacute toxicity study followed OECD 407 guidelines, administering 7.5, 15 and 30 mg/kg/day cyanidin orally to adult SD rats for 28 days.

Acute toxicity testing indicated an LD50 exceeding 300 mg/kg/day with no adverse effects.

Subacute toxicity study demonstrated well-tolerated responses in rats at doses of 7.5–30 mg/kg/day for 28 days.

No significant alterations in organ weights, hematological parameters or liver/kidney functions were observed.

Histopathological examination revealed no adverse findings.

## Graphical abstract

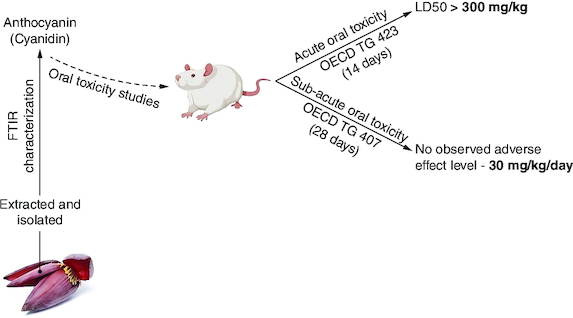

Anthocyanins represent aglycon forms of anthocyanidins and are accountable for the diverse range of colors observed in plants, spanning from orange and red hues to various shades of blue and purple. These water-soluble flavonoids possess a fundamental chemical structure characterized by a flavylium cation, typically comprising one or more sugars along with hydroxyl (-OH) or methoxyl (-OCH_3_) groups. While a comprehensive study of 31 anthocyanidins exists, over 500 distinct anthocyanins have been identified, differing based on hydroxylation, methylation, glycosylation patterns, and variations in acyl substituents attached to the sugar component. Among these, six prevalent anthocyanin types-cyanidin, pelargonidin, peonidin, petunidin, delphinidin and malvidin – are commonly found in fruits and vegetables such as berries, cherries, grape skin, purple sweet potatoes, eggplant, red cabbage, banana bracts and others [1].

Their appeal as medicinal agents stems from various factors, notably their prevalence among the flavonoids commonly consumed in everyday diets, consequently earning recognition for their safety profile. This aspect has led to speculation regarding the potential therapeutic utility of anthocyanins. Given their ready absorption through dietary sources rich in anthocyanins or via supplementation, there exists a hypothesis that these compounds could hold promise in the prevention and management of numerous ailments [2]. To date, comprehensive toxicity investigations specifically focused on anthocyanins are notably absent. Primarily, toxicity assessments have centered around extracts rich in anthocyanins, exemplified by studies utilizing grape skin extract [3], *Dillenia philippinensis* (Rolfe) fruit extract [4], mulberry extract [5] and diverse variants of red flowers [6]. Additionally, research has delved into the acute, subacute and subchronic oral toxicity evaluations of acylation reaction byproducts derived from anthocyanins [7].

Within the European Union (EU), anthocyanins (E 163) are sanctioned as permissible food additives and underwent evaluation by the Scientific Committee on Food (SCF) in 1975 and the Joint FAO/WHO Expert Committee on Food Additives (JECFA) in 1982. Notably, JECFA established an Acceptable Daily Intake (ADI) of 2.5 mg/kg body weight per day specifically for anthocyanins sourced from grape skin; however, the SCF did not designate an ADI for anthocyanins in general. Despite this approval, EU regulations do not delineate the specific fruits and vegetables allowable for generating the food additive anthocyanins. In addition, the European Union directives do not clearly specify the constituents or precise types of anthocyanins present in the food additive E 163. This particular food additive is assigned the European food additive code (E163) for anthocyanins, which are a group of natural pigments responsible for the red, purple and blue colors seen in various fruits, vegetables and flowers. Typically, these substances manifest in food as glycosides or anthocyanins. Fruit extracts, serving as reservoirs of multiple anthocyanin compounds, have predominantly facilitated studies exploring the toxicity kinetics and toxicological properties of anthocyanins. Consequently, these investigations have primarily yielded insights into anthocyanins collectively rather than specific individual compounds. The relevance of the ingredients utilized in various trials remains uncertain concerning the assessment of the specific anthocyanins constituting the food additive E 163, primarily due to the lack of comprehensive characterization of these anthocyanins. As a result, the current toxicological database, as evaluated by the EFSA Panel, is considered inadequate for establishing a precise numerical acceptable daily intake (ADI) for anthocyanins [8]. These circumstances underscore the necessity for conducting an oral-dose toxicity study specifically focusing on anthocyanin compounds.

Moreover, due to their unappealing taste, banana bracts, recognized as a prominent reservoir of anthocyanin content, are often considered as discarded food material despite being derived from one of the extensively cultivated crops in India. Consequently, these bracts might be considered a cost-effective substitute source of anthocyanins, especially in light of the limited availability or absence of alternative sources, such as berry varieties, particularly suited to the Indian population. Anthocyanin-rich extracts obtained from banana bracts have been proved to be a potential source of food colorant [9,10] and various therapeutic activities like antioxidant [11], anti-inflammatory, anti-proliferative [12], and nutritional fiber properties [13]. However, the entirety of this research has been confined to *Musa paradisiaca*. The rationale behind the selection of this specific plant source stemmed from the observation that among the six prevalent commercial banana cultivars cultivated in India, the flower bract of *Musa acuminata* Colla (specifically the red variation within the AAA group) exhibited the highest anthocyanin content, a determination made via UV-visible spectrophotometry [14]. Furthermore, it has been reported that flower bracts exhibit a higher concentration of anthocyanins compared with the red-colored fruit peel [15]. However, none of these studies have focused on purified anthocyanin compounds, nor have they provided a characterization regarding the specific type or quantity of anthocyanins present within the extracts. Moreover, there is an absence of toxicity assessments in any of these studies, particularly regarding the banana bract extracts that are notably rich in anthocyanins.

Previously, we successfully extracted and isolated cyanidin, an anthocyanidin variety, from the outer colored floral bracts obtained from *Musa acuminata* Colla (specifically, the red variant within the AAA cultivar group), and the corresponding findings have been published [16,17]. Moreover, we have effectively characterized the isolated compound, and the corresponding data has been previously published. Peak purity analysis of the isolated cyanidin was conducted via high performance liquid chromatography (HPLC), revealing a purity of 91% compared with the HPLC standard cyanidin, which exhibited a purity of 99%. Additionally, structural characterization was accomplished using proton NMR and LC-MS analysis [16]. The present study aims to assess the safety profile of cyanidin, an anthocyanin, through the conduction of acute oral toxicity and subacute repeated dose oral toxicity evaluations. These assessments were performed following the guidelines outlined in OECD 423 and 407, respectively.

## Materials & methods

### FTIR spectroscopy

Fourier-transform infrared (FT-IR) spectra were obtained using an FT-IR spectrometer (Perkin Elmer FTIR spectrophotometer). The extract was analyzed using FT-IR analysis to identify its functional groups. The specimens were made by compacting them into potassium bromide pellets using pressure. Later on, the samples were placed in the line of light to allow the transmission of infrared light for spectrum analysis. The spectra were measured across the 400–4000 cm^1^ range with a resolution of 4 cm^1^ [18].

### Animal housing & experimental conditions

Adult Sprague-Dawley rats (SD) aged 8 weeks, weighing between 150 and 180 g was procured from the Biogen Laboratory and Animal Facility situated in Bangalore, India. Following procurement, a period of 1 week was dedicated to acclimating the animals to standard laboratory conditions, maintaining a controlled environment of 22 ± 3°C temperature, a 12-h light/dark cycle, and a humidity level of 50 ± 5%. Male and female rats were housed separately in groupings of five per cage, given unlimited access to water and regular rodent chow. Ethical clearance was obtained from the institutional animal ethics committee (IAEC/291/2022) on 12 December 2022.

According to OECD guidelines for acute and subacute oral toxicity studies, animals are euthanized if they exhibit severe toxicity symptoms such as prostration, difficulty breathing, convulsions, significant weight loss, marked behavior changes or irreversible organ dysfunction. Euthanasia is promptly conducted to minimize suffering and adhere to institutional protocols and regulatory standards. In our study, we closely adhered to these guidelines. As none of the animals showed any signs of toxicity, euthanasia was performed at the end point of the studies. However, we were prepared to euthanize them at any point if necessary, following both OECD and institutional guidelines.

All animals were regularly monitored, following the guidelines stipulated by the OECD, which recommend observations at least once daily. Additionally, this monitoring adhered to institutional ethical standards to ensure the welfare of the animals throughout the study. The animals were euthanized using an overdose of anesthesia, specifically thiopental sodium at a dose of 50 mg/kg administered intraperitoneally.

### Toxicity assessment

The study was carried out in accordance with the guidelines set forth by the OECD.

### Acute oral toxicity study

In accordance with OECD 423 recommendations, the acute oral toxicity test was carried out [19]. For the acute oral toxicity study, six adult female SD rats weighing between 150 and 180 g were utilized. Among these, three rats received a single oral dose of 300 mg/kg of the test substance, while the remaining three rats served as the control. As per OECD guidelines, the recommended starting dose of 300 mg/kg body weight was employed due to the absence of prior information on the substance being tested, adhering to animal welfare considerations. Each rat underwent continuous monitoring for mortality and observed behavioral alterations, initially within the first 30 min post-dosing, intermittently over the initial 24-h period (with particular focus on the initial 4 h), and subsequently on a daily basis for a total duration of 14 days.

The animals were given unlimited access to water and regular rodent chow, and meticulous records were maintained encompassing their daily food consumption, water intake, body weight alterations, observations pertaining to mortality and any visible physiological changes. Subsequently, a comprehensive necropsy was performed on all subjects as part of the evaluation process.

### Subacute oral toxicity study

The subacute toxicity investigation was conducted over duration of 28 days in SD rats, in accordance with the guidelines delineated by the OECD protocol 407 [20]. Forty adult SD rats, aged 8 weeks, were divided into four cohorts consisting of ten rats each, comprising five females and five males, out of which one group functioned as the control, receiving standard food and water, while the remaining three groups were administered cyanidin at doses of 30, 15 and 7.5 mg/kg/day, respectively, via oral gavage, sustained over a period of 28 days. Prior to the initial exposure, regular monitoring was done once a day and at least once weekly, thorough clinical examinations were conducted on all subjects. Observations encompassed various parameters including mortality, motor activity, reflexes (righting, corneal, and pineal), escape tendencies as well as physical indicators like pallor, pilo-erection, salivation and presence of diarrhea among the animals.

Upon completion of the study, all animals were euthanized by decapitation on day 29 following an overnight fasting period. Subsequently, a comprehensive necropsy was conducted, involving the liver, spleen, brain, lungs, kidneys, heart, ovaries and testes. Immediately after, these organs were removed, weighed and evaluated.

### Measurement of hematological & biochemical parameters

The retro-orbital bleeding method, following a lateral approach as per established protocols, was utilized to collect blood samples of sufficient volume and quality [21], to obtain blood samples of adequate volume and quality. The blood samples were divided into two halves after collection. To make hematological testing easier, one part was placed into an EDTA aliquot tube, while the other was centrifuged. Serum was extracted from the centrifuged samples in preparation for a further biochemical examination. The Sysmex KX-21 automated blood analyzer, manufactured by Sysmex Corp. (Japan), was utilised to perform an extensive evaluation of many hematological parameters. As part of this, measurements of hemoglobin levels, mean RBC hemoglobin concentration (MCHC), mean corpuscular volume (MCV), mean corpuscular hemoglobin (MCH), total red blood cell (RBC) count, packed cell volume (PCV), platelet count and differential WBC counts were made.

In parallel, blood samples were taken for biochemical tests, with an emphasis on bilirubin levels and liver enzymes including aspartate aminotransferases (AST) and alanine aminotransferases (ALT). Using an automated serum biochemistry analyzer made by Beckman Coulter (Japan), the serum kidney parameters, such as blood urea nitrogen, creatinine, protein levels and serum cholesterol levels, were determined in accordance with the guidelines given. Furthermore, a glucometer was used to measure blood glucose levels by the tail-vein prick technique [22].

### Histopathology evaluation

Tissue sections were preserved in a 10% (v/v) solution of neutrally buffered formalin for 24 h at 4°C. Following this, the brain specimens underwent embedding in paraffin through standard procedures. Serial sections, each measuring 5 μm in thickness and spaced at 100 μm intervals, were obtained. These sections were affixed to slides treated with poly-L-lysine, subsequently subjected to deparaffinization using xylene, gradual rehydration with ethanol, and staining using hematoxylin and eosin (H&E). Examination of tissue morphology was conducted utilizing light microscopy [23].

### Statistical analysis

The statistical analysis was performed utilizing GraphPad Prism version 8.0. Data are presented as mean values along with the standard deviation (SD) unless specifically indicated otherwise. A two-way analysis of variance (ANOVA) was utilized to determine substantial variations among the groups. The predetermined threshold for statistical significance across all computations was established at 0.05. All statistical analyses were conducted using GraphPad Prism software (version 8.0.1 for Windows, CA, USA).

## Results

### Characterization of the isolated anthocyanin using FTIR spectroscopy

**Figure 1.**
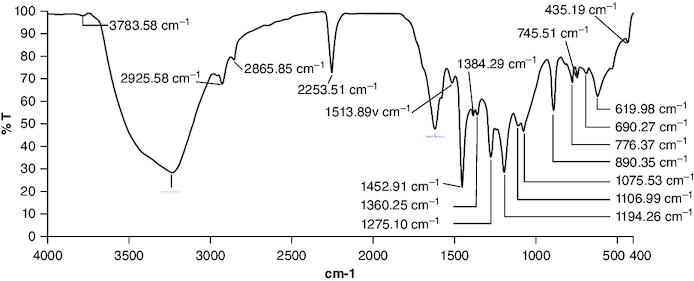
FT-IR spectrum of isolated compound which corresponds with molecular structure of cyanidin.

FT-IR analysis was carried out to confirm the structure of cyanidin by determining its functional groups. Figure 1 shows the FT-IR spectra of the isolated compound. The strong and broad absorption band at 3237 cm^-1^ is attributed to the stretching vibrations of -OH [24] (hydroxyl from the aromatic rings). The absorption bands at 2925, 2865 and 2253 cm^-1^ are referenced to -CH_2_, -CH_3_ and -CH stretching vibrations [25]. The absorption band at 1620 cm^-1^ is attributed to the carbonyl functional group (-C=O stretching), and 1513 cm^-1^ denotes -C=C stretching in the aromatic rings. These results are consistent with the results obtained by Lin *et al.* [26]. The fingerprint region of the spectrum shows an absorption band at 1452 cm^-1^, which corresponds to -CH bending, and also at 1075 cm^-1^, which corresponds to -CO bending vibrations. These findings are comparable to that of Silva *et al.* [27].

**Figure 2.**
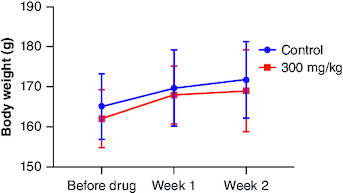
Body weight changes during 14 days of acute oral toxicity test. The data (n = 3) is shown as mean ± standard deviation. Two-way analysis of variance was used for the comparative analyses, and Tukey's test was used for the pairwise post-hoc analysis. A p-value of less than 0.05 was used as the threshold for statistical significance.

**Figure 3.**
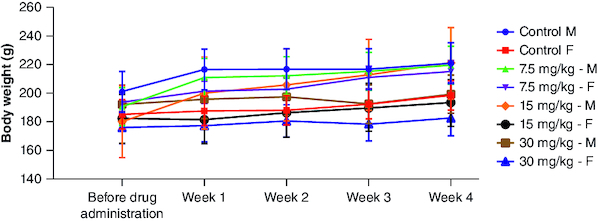
Body weight changes during 28 days of subacute oral toxicity test. The data is shown as mean ± standard deviation (n = 5). Two-way analysis of variance was used for the comparative analyses, and Tukey's test was used for the pairwise post-hoc analysis. A p-value of less than 0.05 was used as the threshold for statistical significance. ‘M’ represents male group and ‘F’ represents female group.

### Acute oral toxicity

Following the oral administration of cyanidin at a single dose of 300 mg/kg for 14-day duration in female subjects, no signs of toxicity or mortality were observed. Comprehensive evaluation of various physiological systems, including respiratory, circulatory, autonomic, central nervous systems, somatomotor activity, ocular functions, mucous membranes, integumentary system and behavioral patterns, did not reveal any indications of toxicity. Additionally, no manifestations of tremors, convulsions, excessive salivation, diarrhea, lethargy, altered sleep patterns or coma were observed in any of the subjects. Upon the conclusion of the study period, no abnormalities were detected during necropsy of the experimental animals in comparison to the control group (Table 1). Gross pathological examination revealed normal organ colors and architecture comparable to those of the control animals. There were no instances of unusual food or water consumption among any of the subjects throughout the study duration. Variations in body weight were observed throughout the investigation period (Figure 2). Throughout the 2-week trial period, all animals showed a consistent gain in body weight. This gain did not exhibit statistical significance when compared with the control group.

**Table 1.** Results of acute toxicity test.

Dose (mg/kg)	Control (F)	Test group (F)	Percentage of mortality	Signs of toxicity
	D/T	D/T		
300 mg/kg	0/3	0/3	0%	None

D: Dead; F: Female rat; T: Total number of rat.

### Subacute oral toxicity study

#### Survival & clinical observations

Three different doses of cyanidin (7.5, 15 and 30 mg/kg) were administered, and no fatalities were observed during the trial. There were no apparent observations or clinical findings regarding the compound's impact on the overall health of the animals postingestion. As expected, over the 28-day trial period, there was no notable decrease in the animals' body weight (Figure 3). The observed variations in weight gain between male and female members within the individual groups could potentially be attributed to gender-specific traits. However, there were no discernible differences between male and female rats in terms of overall body weight or the rate of body weight gain in response to the treatment. Notably, male rats exhibited higher initial body weights and experienced more significant increases in weight compared with female rats. The observed variances were interpreted within the scope of normal biological fluctuations, given that these values align with the increase in body weight, mirroring those observed in the control male subjects. In both female and male rats receiving cyanidin, no discernible impacts on organ weights were noted when compared with the control group (Table 2). Furthermore, no macroscopic pathological findings or histopathological observations associated with the treatment were identified. Every atypical finding was seen as an outcome of spontaneous changes unconnected to the cyanidin exposure.

**Table 2.** Weight of all major organs measured immediately at the end of the study.

Organ weight (g)	Control – M	Control – F	30 mg/kg – M	30 mg/kg – F
Heart	0.51 ± 0.03	0.52 ± 0.03	0.53 ± 0.02^ns^	0.49 ± 0.01^ns^
Lungs	1.8 ± 0.15	1.73 ± 0.05	1.82 ± 0.03^ns^	1.6 ± 0.04^ns^
Kidney	0.59 ± 0.01	0.53 ± 0.01	0.57 ± 0.02^ns^	0.52 ± 0.01^ns^
Liver	2.44 ± 0.22	2.5 ± 0.06	2.33 ± 0.17^ns^	2.31 ± 0.08^ns^
Brain	1.03 ± 0.15	1.33 ± 0.49	1.13 ± 0.05^ns^	0.93 ± 0.11^ns^
Spleen	0.31 ± 0.03	0.28 ± 0.05	0.31 ± 0.06^ns^	0.29 ± 0.05^ns^
Stomach	1.06 ± 0.17	0.95 ± 0.14	1.16 ± 0.05^ns^	0.78 ± 0.19^ns^

Organ weights are expressed as mean ± standard deviation (n = 5). Two-way analysis of variance was used for the comparative analyses, and Tukey's test was used for the pairwise post-hoc analysis.

‘Ns’ indicates that there is no significant difference from the corresponding control group (p > 0.05).

#### Hematological parameters

The impact of subacute oral administration of cyanidin on hematological parameters is summarized in Table 3. The results indicate that there were no substantial alterations in the hematological parameters, as most values fell within the normal ranges reported for rats, in accordance with CPSCEA guidelines [28]. Among male treatment groups, no significant differences were noted in RBC, lymphocyte, monocyte, basophil, platelet count and MCH values (p > 0.05). However, significant differences were observed in Hb and MCHC values for the 7.5 and 15 mg/kg groups in comparison to the male control group, with no significant variance noted for the 30 mg/kg group. In addition, the male dosage group receiving 30 mg/kg had a considerably greater total WBC count than the male control group. Regarding female treatment groups, no significant differences were observed in RBC, Hb, total WBC, neutrophil, basophil, platelet count and MCHC values compared with the control females. However, significant differences were found in PCV (7.5 and 30 mg/kg), lymphocyte (30 mg/kg), eosinophil (15 mg/kg) and MCH (15 mg/kg) values when compared with the control females. These discrepancies cannot be conclusively interpreted as a trend in hematological parameter alterations following cyanidin treatment, as all values remained within the normal range. Moreover, these observations may be influenced by baseline values or the hematological status of the animals before drug administration. Notably, significant differences in hematological parameters between genders were observed, which is deemed biologically reasonable. This comparison was conducted within the same gender, comparing between groups.

**Table 3.** Impact of cyanidin on experimental and control groups on hematological parameters.

Hematology	Control – M	Control – F	7.5 mg/kg – M	7.5 mg/kg – F	15 mg/kg – M	15 mg/kg – F	30 mg/kg – M	30 mg/kg – F
RBC (×10/mm^3^)	8.42 ± 0.60	7.56 ± 0.31	8.13 ± 0.49^ns^	7.76 ± 0.43^ns^	8.16 ± 0.17^ns^	7.73 ± 0.32^ns^	8.55 ± 0.69^ns^	7.60 ± 0.08^ns^
PCV (%)	45.74 ± 0.83	40.60 ± 0.78	44.40 ± 1.22^ns^	38.93 ± 1.39*	44.13 ± 1.01*	39.30 ± 1.3^ns^	44.28 ± 0.84^ns^	39.74 ± 0.40*
Hb (g/dl)	14.76 ± 0.26	11.73 ± 0.38	13.63 ± 1.04*	11.63 ± 0.58^ns^	12.96 ± 0.46****	12.16 ± 1.02^ns^	14.92 ± 0.24^ns^	12.25 ± 0.28^ns^
WBC (×10^3^/mm^3^)	8.26 ± 0.32	8.36 ± 0.58	8.7 ± 0.25^ns^	7.86 ± 0.68^ns^	8.13 ± 0.48^ns^	7.96 ± 0.33^ns^	9.2 ± 0.32**	8.75 ± 1.02^ns^
Neutrophlis (%)	20.10 ± 0.96	18.95 ± 0.90	21.36 ± 1.35^ns^	20.84 ± 0.98^ns^	22.43 ± 1.79***	20.5 ± 0.81^ns^	20.05 ± 0.44^ns^	19.23 ± 0.24^ns^
Lymphocytes (%)	73.66 ± 3.03	71.43 ± 1.14	75.63 ± 3.17^ns^	73.2 ± 2.13^ns^	70.6 ± 0.87^ns^	71.6 ± 2.34^ns^	80.75 ± 1.10^ns^	76.25 ± 1.02**
Eosinophils (%)	2.03 ± 0.55	1.9 ± 0.45	1.26 ± 0.17**	1.66 ± 0.35^ns^	1.96 ± 0.14^ns^	1.13 ± 0.24**	2.20 ± 0.73^ns^	2.05 ± 0.20^ns^
Monocytes (%)	1.06 ± 0.58	1.25 ± 0.89	0.46 ± 0.26^ns^	0.63 ± 0.31*	0.3 ± 0.3^ns^	0.7 ± 0.20^ns^	1.45 ± 1.18^ns^	1.75 ± 0.20^ns^
Basophils (%)	0.60 ± 0.4	0.66 ± 0.17	0.90 ± 0.23^ns^	0.43 ± 0.33^ns^	0.4 ± 0.30^ns^	0.66 ± 0.17^ns^	0.82 ± 0.12^ns^	0.55 ± 0.44^ns^
Platelets (×10^3^/mm^3^)	1048.16 ± 31.22	1161.13 ± 102.40	1032.36 ± 33.94^ns^	1077.96 ± 87.60^ns^	1100.33 ± 84.44^ns^	1069.6 ± 88.50^ns^	1185.7 ± 45.07^ns^	1090.25 ± 127.41^ns^
MCV (fL)	54.44 ± 1.27	56.92 ± 1.42	54.50 ± 1.50^ns^	53.66 ± 0.73***	53.13 ± 0.89^ns^	55.46 ± 1.50^ns^	54.63 ± 1.43**	56.06 ± 1.53^ns^
MCH (pg)	18.75 ± 0.68	18.33 ± 0.29	18.6 ± 0.47^ns^	18.73 ± 1.26^ns^	18.76 ± 0.38^ns^	20.13 ± 0.38**	19.06 ± 0.40^ns^	18.50 ± 0.72^ns^
MCHC (g/dl)	33.93 ± 0.38	34.83 ± 1.09	32.06 ± 0.86*	32.56 ± 0.61^ns^	31.53 ± 1.10**	34.1 ± 0.96^ns^	34.13 ± 1.92^ns^	34.73 ± 1.74^ns^

The data is expressed as mean ± standard deviation (n = 5). Tukey's test was used in pairwise post-hoc analysis after a two-way analysis of variance was used for statistical comparisons. ‘ns’ indicates that there is no significant difference from the corresponding control group (p > 0.05), while *denotes a statistically significant difference (p < 0.05) from the corresponding control. Additionally, **indicates p < 0.01; ***indicates p < 0.001; and ****denotes p < 0.0001.

#### Biochemical parameters

Table 4 summarizes the impact of subacute oral administration of cyanidin on liver function. In male treatment groups, no significant differences were noted in SGPT and bilirubin values (p > 0.05). However, a significant difference was observed in SGOT values in the high-dose male group. Similarly, in the 15 mg/kg female group, a significant difference was observed in comparison to the control females. Notably, these statistically significant values fall within the normal range of rat biochemistry according to CPSCEA guidelines [28]. Moreover, all other liver function test (LFT) values remain statistically insignificant when compared with the corresponding control group.

**Table 4.** Impact of cyanidin on experimental and control groups on liver function.

LFT	Control – M	Control – F	7.5 mg/kg – M	7.5 mg/kg – F	15 mg/kg – M	15 mg/kg – F	30 mg/kg – M	30 mg/kg – F
SGOT (U/l)	73.66 ± 3.40	69.80 ± 1.88	74.13 ± 1.58^ns^	70.12 ± 1.61^ns^	73.61 ± 2.48^ns^	68.53 ± 1.72^ns^	71.68 ± 1.45*	69.86 ± 4.73^ns^
SGPT (U/l)	29.60 ± 1.02	28.06 ± 1.81	29.93 ± 1.11^ns^	27.34 ± 0.60^ns^	28.43 ± 2.02^ns^	26.53 ± 1.26**	29.22 ± 1.62^ns^	27.26 ± 0.41^ns^
Bilirubin (mg/dl)	0.36 ± 0.04	0.38 ± 0.10	0.31 ± 0.04^ns^	0.32 ± 0.02^ns^	0.30 ± 0.031^ns^	0.35 ± 0.01^ns^	0.44 ± 0.13^ns^	0.36 ± 0.08^ns^

The information is shown as mean ± standard deviation (n = 5). Two-way analysis of variance was used for the comparative analyses, and Tukey's test was used for pairwise post-hoc analysis. A statistically significant difference (p < 0.05) is denoted by *p < 0.01 is represented by **while ‘ns’ indicates no significant difference (p > 0.05) compared with the corresponding control group.

The impact of subacute oral administration of cyanidin on renal function is summarized in Table 5. Total protein and globulin levels did not exhibit statistically significant differences in both male and female treatment groups when compared with their respective control groups (p > 0.05). However, notable differences were observed: albumin levels showed a statistically significant difference in the 30 mg/kg treated male group, while BUN levels displayed significance in the 7.5 and 15 mg/kg male groups, as well as the 30 mg/kg female group compared with their respective controls (p < 0.05). Similarly, creatinine levels showed statistically significant differences in both male and female 15 mg/kg treated groups when compared with their respective controls (p < 0.05).

**Table 5.** Effect of cyanidin on renal function of control and experimental groups.

RFT	Control – M	Control – F	7.5 mg/kg – M	7.5 mg/kg – F	15 mg/kg – M	15 mg/kg – F	30 mg/kg – M	30 mg/kg – F
Protein (g/dl)	7.23 ± 0.48	6.16 ± 0.37	7.22 ± 0.17^ns^	6.56 ± 0.33^ns^	7.23 ± 0.20^ns^	6.96 ± 0.63^ns^	7.03 ± 0.29^ns^	6.83 ± 0.40^ns^
Albumin (g/dl)	4.46 ± 0.38	3.73 ± 0.56	4.63 ± 0.29^ns^	3.96 ± 0.48^ns^	4.36 ± 0.31^ns^	3.76 ± 0.26^ns^	3.56 ± 0.33**	3.93 ± 0.34^ns^
Globulin (g/dl)	3.46 ± 0.35	3.36 ± 0.32	3.23 ± 0.23^ns^	3.53 ± 0.58^ns^	3.16 ± 0.20^ns^	3.32 ± 0.26^ns^	3.53 ± 0.37^ns^	3.66 ± 0.59^ns^
BUN (mg/dl)	18.46 ± 1.26	19.33 ± 0.64	19.36 ± 0.55*	19.34 ± 0.26^ns^	19.16 ± 0.43*	19.26 ± 0.52^ns^	18.66 ± 1.72^ns^	18.33 ± 1.41**
Creatinine (mg/dl)	0.83 ± 0.12	0.73 ± 0.03	0.83 ± 0.06^ns^	0.76 ± 0.03^ns^	0.93 ± 0.03*	0.83 ± 0.13*	0.80 ± 0.09^ns^	0.82 ± 0.11^ns^

The data (n = 5) is shown as mean ± standard deviation. Tukey's test was used in pairwise post-hoc analysis after a two-way analysis of variance was used for the comparative analyses. A statistically significant difference (p < 0.05) is denoted by *p < 0.01 is represented by **while ‘ns’ indicates no significant difference (p > 0.05) compared with the corresponding control group.

The impact of subacute oral administration of cyanidin on serum cholesterol and blood glucose levels is outlined in Table 6. Blood glucose levels displayed significant differences across all treatment groups regardless of gender or dosage when compared with their respective controls. However, serum cholesterol levels did not show a consistent pattern corresponding to the administered dose of cyanidin. Notably, the high-dose cyanidin group did not exhibit any significant difference in cholesterol levels compared with the control groups. Both cholesterol and glucose levels displayed some fluctuations compared with the controls following drug administration. However, these variations did not deviate from the normal range. These fluctuations might be attributed to variations in baseline values, thus accounting for inter-individual differences among rats.

**Table 6.** Effect of cyanidin on serum cholesterol and blood glucose levels of control and experimental groups.

Other parameters	Control – M	Control – F	7.5 mg/kg – M	7.5 mg/kg – F	15 mg/kg – M	15 mg/kg – F	30 mg/kg – M	30 mg/kg – F
Cholesterol (mg/dl)	123.2 ± 17.51	111.2 ± 8.97	132.73 ± 4.01**	110.53 ± 1.60^ns^	129.4 ± 9.61*	125.13 ± 8.48*	122.63 ± 19.33^ns^	113.63 ± 11.30^ns^
Glucose (mg/dl)	112.23 ± 28.70	119.63 ± 13.80	108.66 ± 4.71*	102.86 ± 4.09***	105.43 ± 8.66**	113.06 ± 11.10*	124.24 ± 30.36***	117.46 ± 22.04*

A mean ± standard deviation (n = 5) is used to represent the data. Pairwise post-hoc analysis using Tukey's test was performed after a two-way analysis of variance was used for the comparative analyses. ‘ns’ indicates that there is no significant difference from the corresponding control group (p > 0.05), while *denotes a statistically significant difference (p < 0.05) from the corresponding control. Additionally, **indicates p < 0.01, and ***indicates p < 0.001.

#### Histopathological examination

**Figure 4.**
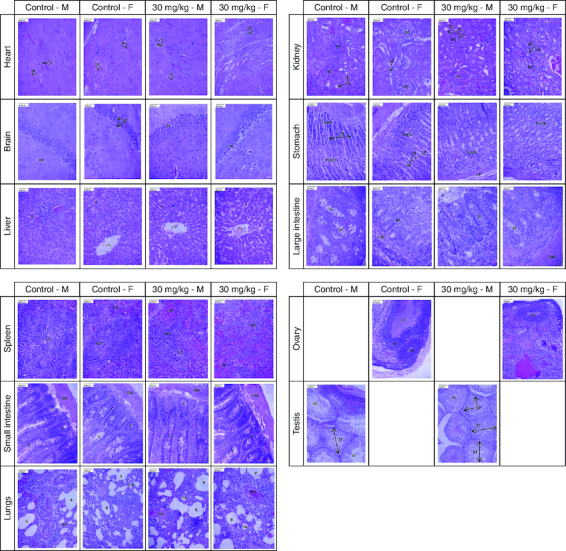
Presents photomicrographs showcasing significant organs from both genders in the high-dose and control groups. Staining was performed using hematoxylin and eosin, with a scale bar set at 800 μm. Morphology of all major organs of treated groups represents normal architecture comparable to that of control groups. **Heart** – MF: Myocardial fiber; N: Nucleus; **Brain** – GL: Granular layer; ML: Molecular layer; PN: Pyramidal neuron; **Liver** – CV: Central vein; S: Sinusoid; **Kidney** – PCT: Proximal convoluted tubule; RC: Renal corpuscle; RT: Renal tubule; **Stomach** – Ccell: Chief cell; GP: Gastric pit; LP: Lamina propria; Pcell: Parietal cell; **Large intestine** – IG: Intestinal gland; GC: Goblet cell; L: Lumen; M: Mucosa; SM: Submucosa; **Spleen** – CA: Central arteriole; RP: Red pulp; WP: White pulp; **Small intestine** – C: Crypt; SM: Submucosa; V: Villi; **Lungs** – B: Bronchiole; BV: Blood vessel; PA: Pulmonary alveoli; **Ovary** – G: Granulosa cell; GF: Graafian follicle; OC: Oocyte; **Testis** – SP: Spermatocyte; ST: Seminiferous tubule; SZ: Spermatozoa.

The histopathological examination of major organs following the administration of 30 mg/kg of cyanidin for a duration of 28 days revealed normal architectural features that were comparable to those observed in the control group (Figure 4).

## Discussion

Given that pharmaceutical medications are known to have significant side effects, food-derived bioactive molecules have attracted interest as possible regulators against a range of chronic illnesses due to their relatively low toxicity. Developing new dietary treatments based on various bioactive ingredients found in food has become a viable strategy for treating a variety of illnesses. Red cabbage, blueberries, blackcurrants, mulberries, cherries, black elderberries, black soybeans, chokeberries and jaboticaba peel are major sources of anthocyanins, a subclass of flavonoids. These sources are rich in anthocyanins, which include cyanidins, peonidins, petunidins, delphinidins and malvidins [29]. Due to their widespread consumption, anthocyanins have been the subject of extensive research to elucidate their various biological activities. Anthocyanins have demonstrated multifaceted biological activities, including documented evidence supporting their anti-obesity [30], antioxidant [31], anti-inflammatory [32], anti-diabetic [33,34] and neuroprotective activities against neurodegenerative diseases like Alzheimer's disease [1,16]. Additionally, anthocyanins sourced from diverse origins are recognized for their potential as natural food colorants [35]. Given the broad spectrum of activity exhibited by this phytocompound against various illnesses, it becomes imperative to evaluate its safety profile. Establishing a maximum safe dose is crucial to guide the consumption of anthocyanin and prevent potential adverse health effects. The present study provides pivotal data for regulatory compliance. Regulatory bodies have not yet established a definitive safe dose for anthocyanin due to the absence of comprehensive toxicity studies specifically on purified anthocyanin. Previous toxicity investigations have primarily focused on anthocyanin-rich extracts, which may not provide reliable data as these extracts often contain numerous other phytocompounds that could potentially exert either adverse or beneficial effects, rendering the findings less conclusive for establishing safety guidelines.

The investigation led by Yang *et al.* encompassed a comprehensive assessment of acute, subacute and subchronic toxicity regarding anthocyanin-derived acylation reaction products, particularly focusing on anthocyanin-lauric acid derivatives (ALDs). Notably, acute toxicity evaluations in mice revealed an LD50 value of ALDs exceeding 10 g/kg. Subsequent sub-chronic toxicity analyses demonstrated that an intake below 0.60 g/kg body weight did not induce adverse effects in terms of mortality, body weight, food and water consumption, gross pathology, histology, hematology or serum biochemistry. The acylation of anthocyanin in this study aimed to enhance its stability. The research contributes significant insights into the toxicity profile of anthocyanin derived from black rice (*Oryza Sativa* L.). It is essential to acknowledge, although, that the research employed a combination of anthocyanins, mostly consisting of cyanidin-3-glucoside, in addition to additional anthocyanins such cyanidin-3,5-diglucoside, cyanidin-3-rutinoside, peonidin-3-glucoside and peonidin-3-rutinoside. However, a weakness in the research was that the study did not include individual characterizations of these substances [7].

In a study conducted by Bagchi *et al.*, the acute dermal toxicity of anthocyanin-rich extracts derived from six distinct edible berries was examined, revealing an acute-dermal LD50 value surpassing 5 g/kg. However, a significant limitation within the study lies in the absence of characterization or identification of the extract's constituents to discern or elucidate the structural composition of anthocyanins. The results provide support for the safety profile of edible berries, indicating tolerability at doses beyond 5 g/kg. Nevertheless, it is crucial to consider that while berries are recognized as abundant sources of anthocyanins, the applicability and reliability of these findings in defining the toxicity profile of anthocyanin may be constrained. This limitation emanates from the use of extracts originating from six different berries, thereby raising uncertainties about the primary type or composition of anthocyanin predominantly present within the extract [36]. In their research, Hong *et al.* conducted a 90-day subchronic oral toxicity evaluation using mulberry extract, recognized for its high anthocyanin content. The study established a No-Observed Adverse Effect Level (NOAEL) of 4200 mg/kg, equivalent to 1058.5 mg/kg of anthocyanin dosage. Analysis of the mulberry extract revealed that anthocyanins accounted for 25.2% of the total extract weight. Importantly, the toxicity assessment encompassed the entirety of the mulberry extract, and conclusions regarding the toxicity profile of anthocyanin were inferred from the comprehensive toxicity evaluation of the extract, taking into account the identified proportion of anthocyanin within the extract [5].

To date, there is a noticeable absence of studies specifically focusing on purified anthocyanins derived from natural sources. However, our investigation provides a crucial safety assessment of cyanidin, recognized as the most prevalent anthocyanin in nature. It is important to note that while our findings offer insights into the safety considerations pertaining to cyanidin, they may not be extrapolated to other anthocyanins such as delphinidins, malvidins, pelargonidins, peonidins and petunidins. The outcomes of this study serve as foundational evidence for future endeavors aimed at exploring the therapeutic potential mediated by anthocyanins. Furthermore, they lay the groundwork for potential subacute therapy spanning 28 days. Nonetheless, it is highly recommended that chronic toxicity studies be conducted to comprehensively evaluate the safety profile of cyanidin following administration over a duration of at least 3 months. Such studies are essential to discern any potential late-onset reactions that might manifest over an extended period post-administration.

## Conclusion

In summary, the single oral administration of 300 mg/kg of cyanidin over a 14-day period did not manifest any acute toxicity effects, indicating an LD50 value greater than 300 mg/kg. Furthermore, there were no significant side effects from the daily oral administration of cyanidin for 28 days in a row at dosages of 30, 15 and 7.5 mg/kg. As a result, the No-Observed-Adverse-Effect Level (NOAEL) for cyanidin was established at 30 mg/kg/day.

## References

[1] Suresh S, Begum RF, Singh SA, Vellapandian C. Anthocyanin as a therapeutic in Alzheimer's disease: a systematic review of preclinical evidences. Ageing Res. Rev. 76, 101595 (2022). 35217244 10.1016/j.arr.2022.101595

[2] Winter AN, Bickford PC. Anthocyanins and their metabolites as therapeutic agents for neurodegenerative disease. Antioxidants 8(9), 333 (2019). 31443476 10.3390/antiox8090333 PMC6770078

[3] Bentivegna SS, Whitney KM. Subchronic 3-month oral toxicity study of grape seed and grape skin extracts. Food Chem. Toxicol. 40(12), 1731–1743 (2002). 12419686 10.1016/s0278-6915(02)00155-2

[4] Barcelo RC, Jonathan MB, Rosuman PF et al. Preliminary *In vivo* evaluation of the acute toxicity of Dillenia philippinensis (Rolfe) fruit extract, anthocyanins and polyphenols in Mice (Mus musculus). Int. J. Biosci. 10(05), 51–65 (2017).

[5] Hong M, Lu M, Qian Y et al. A 90-day sub-chronic oral toxicity assessment of mulberry extract in Sprague Dawley rats. Inquiry (United States) 58, 00469580211056044 (2021). 10.1177/00469580211056044 PMC8613892 34812659

[6] Harlinda K, Rosiarto AM, Putri AS et al. Antioxidant and toxicity properties of anthocyanin extracted from red flower of four tropical shrubs. Nisantara Biosci. 8(2), 135–140 (2016).

[7] Yang X, Sun H, Tu L et al. Investigation of acute, subacute and subchronic toxicities of anthocyanin derived acylation reaction products and evaluation of their antioxidant activities *in vitro*. Food Funct. 11(12), 10954–10967 (2020). 33283810 10.1039/d0fo01478h

[8] Aguilar F, Crebelli R, Dusemund B et al. Scientific Opinion on the re-evaluation of anthocyanins (E 163) as a food additive. EFSA J. 11(4), 3145 (2013).

[9] Alexandra Pazmio-Durán E, Giusti MM, Wrolstad RE et al. Anthocyanins from banana bracts (Musa X paradisiaca) as potential food colorants. Food Chem. 73(3), 327–332 (2001).

[10] Chiang SH, Yang KM, Lai YC et al. Evaluation of the *in vitro* biological activities of Banana flower and bract extracts and their bioactive compounds. Int. J. Food Prop. 24(1), 1–16 (2021).

[11] Sujithra S, Manikkandan TR. Extraction of anthocyanin from banana (Musa paradisiaca) flower bract and analysis of phytochemicals, antioxidant activities and anthocyanin content. J. Chem. Pharmaceut. Sci. 12(3), 102–104 (2019).

[12] Susaritha R, Prakash A, Vadivel V. Utilization of anthocyanins-rich extract from banana bract in the green synthesis of AgNPs with anti-proliferative potential. Proc. Natl Acad. Sci. India Sect. B – Biol. Sci. 91(2), 397–406 (2021).

[13] Begum YA, Deka SC. Ultrasound-assisted extracted dietary fiber from culinary banana bract as matrices for anthocyanin: its preparation, characterization and storage stability. J. Food Sci. Technol. 57(6), 2354–2363 (2020). 32431361 10.1007/s13197-020-04273-0 PMC7230098

[14] Preethi P, Balakrishnamurthy G. Assessment of banana cultivars for pigment extraction from bracts, its suitability and stability as food colourant. Internat. J. Proc. & Post Harvest Technol. 2(2), 98–101 (2011).

[15] Rosalina Y, Warsiki E, Fauzi AM et al. Study of anthocyanin extraction from red banana (Musa sapientum L. var Rubra) waste and characteristics of light effects. Sci. Technol. Indonesia 7(4), 522–529 (2022).

[16] Suresh S, Vellapandian C. Cyanidin ameliorates bisphenol a-induced Alzheimer's disease pathology by restoring Wnt/β-catenin signaling cascade: an *in vitro* study. Mol. Neurobiol. 1, 1–17 (2023). 10.1007/s12035-023-03672-6 37843801

[17] Suresh S, Vellapandian C. Restoring impaired neurogenesis and alleviating oxidative stress by cyanidin against bisphenol a-induced neurotoxicity: *in vivo* and *in vitro* evidence. Curr. Drug Discov. Technol. doi:10.2174/0115701638280481231228064532 (2024) (Online ahead of print). 38279724

[18] Manzoor MF, Zeng XA, Rahaman A et al. Combined impact of pulsed electric field and ultrasound on bioactive compounds and FT-IR analysis of almond extract. J. Food Sci. Technol. 56(5), 2355 (2019). 31168118 10.1007/s13197-019-03627-7 PMC6525683

[19] OECD. Test no. 423: acute oral toxicity – acute toxic class method. Oecd Guidel. Test. Chem. 1–14 (2002). Available from: doi:10.1787/9789264071001-en

[20] OECD. Test no. 407: repeated dose 28-day oral toxicity study in rodents. Oecd. Guidel. Test. Chem. (2008). Available from: doi:10.1787/9789264070684-en

[21] Parasuraman S, Raveendran R, Kesavan R. Blood sample collection in small laboratory animals. J. Pharmacol. Pharmacother. 1(2), 87 (2010). 21350616 10.4103/0976-500X.72350 PMC3043327

[22] Zhang C, Zhang D, Wang H et al. Hyperbaric oxygen treatment improves pancreatic β-cell function and hepatic gluconeogenesis in STZ-induced type-2 diabetes mellitus model mice. Mol. Med. Rep. 25(3), 90 (2022). 35039874 10.3892/mmr.2022.12606 PMC8809048

[23] Cardiff RD, Miller CH, Munn RJ. Manual hematoxylin and eosin staining of mouse tissue sections. Cold Spring Harb. Protoc. 2014(6), 655–658 (2014). 24890205 10.1101/pdb.prot073411

[24] Cai J, Zeng F, Zheng S et al. Preparation of lipid-soluble bilberry anthocyanins through acylation with cinnamic acids and their antioxidation activities. J. Agric. Food Chem. 68(28), 7467–7473 (2020). 32551628 10.1021/acs.jafc.0c01912

[25] Liu J, Zhuang Y, Hu Y et al. Improving the color stability and antioxidation activity of blueberry anthocyanins by enzymatic acylation with p-coumaric acid and caffeic acid. LWT 130, 109673 (2020).

[26] Lin Y, Li C, Shao P et al. Enzymatic acylation of cyanidin-3-O-glucoside in raspberry anthocyanins for intelligent packaging: improvement of stability, lipophilicity and functional properties. Curr. Res. Food Sci. 5, 2219–2227 (2022). 36419743 10.1016/j.crfs.2022.11.008 PMC9676150

[27] da Silva HR, de Assis D da C, Prada AL et al. Obtaining and characterization of anthocyanins from Euterpe oleracea (açaí) dry extract for nutraceutical and food preparations. Rev. Bras. Farmacogn. 29(5), 677–685 (2019).

[28] Compendium of CPCSEA. Acts, rules and guidelines: committee for the purpose of control and supervision of experiments on animals (2018). Available from: https://ccsea.gov.in/WriteReadData/userfiles/file/Compendium%20of%20CPCSEA.pdf

[29] Lee YM, Yoon Y, Yoon H et al. Dietary anthocyanins against obesity and inflammation. Nutrients 9(10), 1089 (2017). 28974032 10.3390/nu9101089 PMC5691706

[30] Santamarina AB, Calder PC, Estadella D et al. Anthocyanins ameliorate obesity-associated metainflammation: preclinical and clinical evidence. Nutr. Res. 114, 50–70 (2023). 37201432 10.1016/j.nutres.2023.04.004

[31] Zhao X, Yuan Z. Anthocyanins from Pomegranate (Punica granatum L.) and their role in antioxidant capacities *in vitro*. Chem. Biodivers. 18(10), e2100399 (2021). 34388293 10.1002/cbdv.202100399

[32] Morais CA, de Rosso VV, Estadella D et al. Anthocyanins as inflammatory modulators and the role of the gut microbiota. J. Nutr. Biochem. 33, 1–7 (2016). 27260462 10.1016/j.jnutbio.2015.11.008

[33] Rózańska D, Regulska-Ilow B. The significance of anthocyanins in the prevention and treatment of type 2 diabetes. Adv. Clin. Exp. Med. 27(1), 135–142 (2018). 29521054 10.17219/acem/64983

[34] Kim JN, Han SN, Kim HK. Anti-inflammatory and anti-diabetic effect of black soybean anthocyanins: data from a dual cooperative cellular system. Molecules 26(11), 3363 (2021). 34199668 10.3390/molecules26113363 PMC8199741

[35] Roy S, Rhim JW. Anthocyanin food colorant and its application in pH-responsive color change indicator films. Crit. Rev. Food Sci. Nutr. 61(14), 2297–2325 (2021). 32543217 10.1080/10408398.2020.1776211

[36] Bagchi D, Roy S, Patel V et al. Safety and whole-body antioxidant potential of a novel anthocyanin-rich formulation of edible berries. Mol. Cell. Biochem. 281(1–2), 197–209 (2006). 16328973 10.1007/s11010-006-1030-6

